# Massive Transfusion Protocol Activation Does Not Result in Preferential Use of Older Red Blood Cells

**DOI:** 10.1155/2014/328967

**Published:** 2014-09-10

**Authors:** Lauren M. McDaniel, Darrell J. Triulzi, James Cramer, Brian S. Zuckerbraun, Jason L. Sperry, Andrew B. Peitzman, Jay S. Raval, Matthew D. Neal

**Affiliations:** ^1^University of Pittsburgh School of Medicine, Pittsburgh, PA 15261, USA; ^2^Department of Pathology, University of Pittsburgh, Pittsburgh, PA 15213, USA; ^3^The Institute for Transfusion Medicine, Pittsburgh, PA 15213, USA; ^4^Division of Trauma and General Surgery, Department of Surgery, University of Pittsburgh, Pittsburgh, PA 15213, USA; ^5^Department of Pathology and Laboratory Medicine, University of North Carolina, Chapel Hill, NC 27599, USA

## Abstract

Widespread, anecdotal belief exists that patients receiving massive transfusion, particularly those for whom a massive transfusion protocol (MTP) is activated, are more likely to receive older red blood cells (RBCs). Retrospective review of blood bank records from calendar year 2011 identified 131 patients emergently issued ≥10 RBC units (emergency release (ER)) prior to obtaining a type and screen. This cohort was subclassified based on whether there was MTP activation. For comparison, 176 identified patients transfused with ≥10 RBC units in a routine fashion over 24 hours represented the nonemergency release (nER) cohort. Though the median age of ER RBCs was 5 days older than nER RBCs (ER 20, nER 15 days, *P* < 0.001), both fell within the third week of storage. Regardless of MTP activation, transfused ER RBCs had the same median age (MTP 20, no-MTP 20 days, *P* = 0.069). In the ER cohort, transition to type-specific blood components increased the median age of transfused RBC units from 17 to 36 days (*P* < 0.001). These data refute the anecdotal belief that MTP activation results in transfusion of older RBCs. However, upon transition to type-specific blood components, the age of RBCs enters a range in which it is hypothesized that there may be a significant effect of storage age on clinical outcomes.

## 1. Background

The influence of duration of storage of red blood cells (RBCs) on clinical outcomes is currently unknown. Multiple retrospective and observational studies suggest that transfusion of older RBCs, particularly in trauma patients, is associated with increased morbidity including multiple organ failure and nosocomial infection [1–6]. However, the inherent limitations of the study designs of these analyses weaken the conclusions. Furthermore, other authors contend that there is no increase in complication rates attributable to RBC duration of storage [7–10].

The age of stored RBCs in massive transfusion has received particular interest. Most blood banks in the United States practice a “first-in-first-out” inventory policy, which could potentially result in the delivery of large volumes of older RBCs to massively hemorrhaging patients [11–13]. Some have hypothesized that patients receiving massive transfusion, particularly trauma patients, are more likely to receive older RBCs than other patients requiring transfusion [4]. Despite the concern regarding the age of RBCs transfused to patients with massive hemorrhage, there are no studies specifically designed to address whether the use of a massive transfusion protocol (MTP) results in the delivery of older RBCs. Furthermore, although studies exist to suggest that certain ABO blood types may be stored for longer duration due to infrequent use [11, 13], there is no data to suggest how ABO blood type impacts the age of RBCs given in massive transfusion. Our goal was to characterize the age and ABO type of RBCs utilized during massive transfusion at a large, tertiary referral academic medical center. We hypothesized that MTP activation would not result in the transfusion of older RBCs.

## 2. Materials and Methods

A retrospective analysis was performed using blood bank emergency release (ER) and massive transfusion records at the University of Pittsburgh Medical Center and the Institute for Transfusion Medicine from January 1, 2011, through December 31, 2011. This research was conducted after approval by the University of Pittsburgh Medical Center Quality Assurance Committee (QIRB878).

An ER was defined as a request for the immediate release of RBCs in any quantity. This included the issuing of type O units (and not type-specific units) in situations in which there was insufficient time to obtain a patient sample for performance of a type and screen and to release type-specific units. At our institution, at the time of this study, a physician in any location of the hospital had the option to emergently order a massive transfusion of RBCs, defined as ≥10 units in a 24-hour period, with or without activation of the massive transfusion protocol (MTP) described previously [14]. Patients who had ≥10 RBC units issued to them in a single release were identified from the ER records and represented the ER cohort of this study. Using information from the electronic medical records, patients with an issuance of ≥10 RBC units were further categorized based on whether the MTP was or was not activated during their care (MTP or no-MTP subgroups, resp.). Both the ER cohort and the MTP subgroup included patients with traumatic and nontraumatic sources of massive hemorrhage.

Additional data were collected from University of Pittsburgh Medical Center records to identify all patients in 2011 who received massive transfusion. In addition to the ER cohort of patients, this dataset included those who had met massive transfusion criteria but instead received exclusively type-specific products since they had valid type and screens at the time of RBC requests. The majority of these patients were individuals with postsurgical complications and medically bleeding patients who met the criteria for massive transfusion through the serial transfusion of ≥10 RBCs over 24 hours as opposed to an up-front single request. This group represented the nonemergency release (nER) cohort. Importantly, the physicians caring for patients in this group had not requested an ER or activated the MTP at any point, suggesting that they may not have anticipated that the patient would require massive transfusion at the time of initial assessment and RBC infusion. This nER cohort of patients was chosen as a comparison group to the ER patient cohort since patients in both groups had similar RBC transfusion needs.

After identification of the patients within the ER and nER cohorts, an Institute for Transfusion Medicine blood bank database was queried to determine the age of each RBC unit transfused into each patient during the 24-hour period of massive transfusion. The age of each unit was defined as the difference in time between the collection and transfusion date. Additionally, the ABO type of each unit and of each patient was recorded. The vast majority (>95%) of RBCs at our institution have a 42-day shelf life. Of note, universal leukoreduction is not employed at our institution, but rather patients are selectively given leukoreduced RBCs for either prevention of febrile nonhemolytic transfusion reactions, need for CMV risk reduction, or decreasing likelihood of HLA sensitization.

Patients in the ER cohort initially received type O RBCs during their resuscitation. Once the results of their type and screens were known, ABO type-specific units were transfused. However, the point at which the patients began to receive type-specific RBCs could only be determined for non-type O patients due to limitations in the dataset.

Lastly, to estimate the distribution and availability of the different ABO type RBCs available to the patients at our medical center, the regional blood donor center records were analyzed over a 30-year period. Between 1980 and 2010, deidentified records of unique individuals making a donation were analyzed and the ABO type of each donor was recorded.

Statistical analyses were performed using SPSS software, versions 22 (SPSS, Inc.). Mann-Whitney *U* or ANOVA tests were used to compare continuous variables and chi-square tests were used for categorical variables. Statistical significance was defined as *P* < 0.05.

## 3. Results

### 3.1. ER Cohort

**Figure 1:**
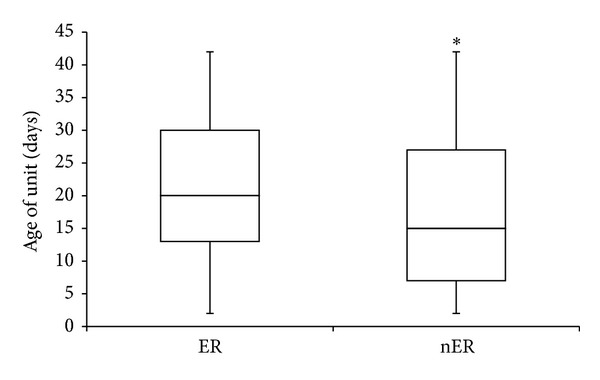
Difference in unit age between ER and nER cohorts. ER, emergency release; nER, nonemergency release; *P < 0.001.

**Figure 2:**
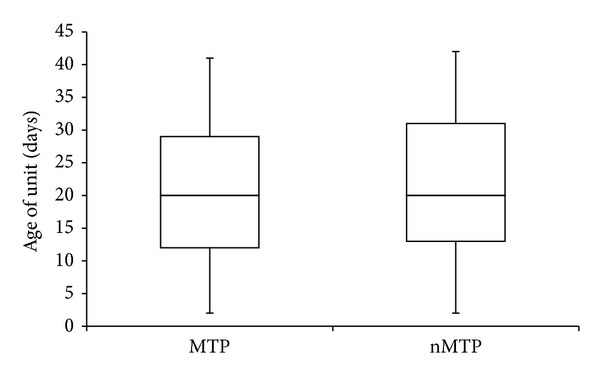
Difference in unit age between MTP and nMTP cohorts. MTP, massive transfusion protocol; nMTP, no massive transfusion protocol; *P* = 0.069.

In the ER cohort, 1500 units of RBCs were transfused into 131 patients. The median RBC age at time of transfusion of these emergently released units was 20 days [IQR 13, 30] (Figure 1). A total of 779 of these ER units were used in MTP activations. Regardless of whether RBCs were requested with or without MTP activation, the median ages of transfused units between the two subgroups were identical with similar interquartile ranges (MTP 20 [IQR 12, 29], nMTP 20 [IQR 13, 31], *P* = 0.069) (Figure 2).

The number of transfused units that were >28 and 35 days was calculated for each patient. In the ER cohort, the median number of units >28 days and 35 days was 2 (IQR 0, 1) and 0 (IQR 0, 0) units, respectively. In the MTP subgroup, the median number of units of each age transfused per patient was 3 (IQR 0, 6) and 0 (IQR 0, 3.5). In the nMTP subgroup, the median number of units >28 days and 35 days was 1 (IQR 0, 3) and 0 (IQR 0, 3) units, respectively. The number of patients who received ≥1 and 5 units ≥28 and 35 days old was calculated (Table 1). There were no statistically significant differences between groups except for the percentage of patients receiving ≥1 unit older than 28 days, which was higher in the MTP group (*P* = 0.02) (Table 1).

**Figure 3:**
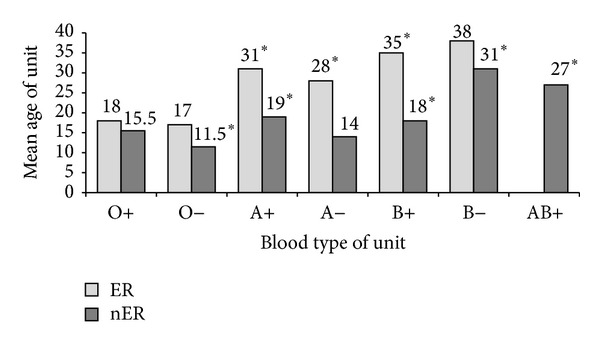
Median age of RBC units categorized by ABO type of unit. Storage ages of RBCs were computed for all transfused units as divided by ABO type. ER, emergency release; nER, nonemergency release; O+, O-positive; O−, O-negative, A+, A-positive; A−, A-negative; B+, B-positive; B−, B-negative; AB+, AB-positive; *P < 0.05 when compared to O+ group.

**Figure 4:**
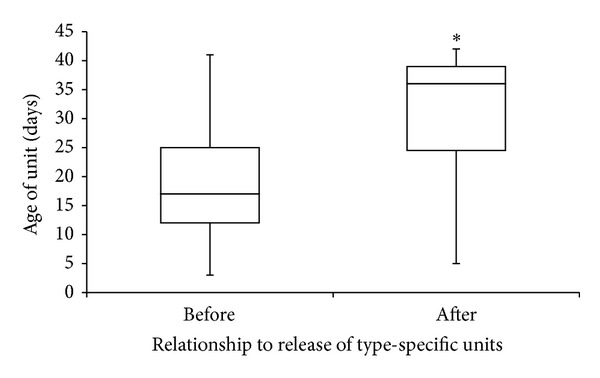
Difference in unit age before and after receiving type-specific units in non-type O patients. *P < 0.001.

When the 1500 transfused RBC units were stratified by ABO type, there were 1180 O-positive, 41 O-negative, 233 A-positive, 7 A-negative, 38 B-positive units, and 1 B-negative unit. Patients in the ER cohort who received type O RBCs received younger units than those who received non-type O blood (Figure 3), as the median age of an O-positive unit was 18 days and the median age of an O-negative unit was 17 days. The median ages of each of the other non-type O units were ≥28 days. Additionally, when the 81 patients in the ER cohort with non-type O blood groups were switched from type O RBCs to type-specific RBCs, the median ages of the transfused units significantly increased by over 2 weeks (17 [IQR 12, 25] days to 36 [IQR 24.5, 39] days; *P* < 0.001) (Figure 4). The average number of type O units transfused in these patients (with blood types other than O) prior to the switch to type-specific units was 8.5 units.

### 3.2. nER Cohort

In the nER cohort, 2849 units were transfused into 176 patients. The median RBC age at time of transfusion of these nonemergently released units was 15 [IQR 7, 27] days. The median number of units older than 28 and 35 days transfused per patient was 2 (IQR 0, 5) and 0 (IQR 0, 3) units, respectively. Importantly, there were no statistically significant differences in the percentage of patients receiving ≥1 or 5 units ≥28 or 35 days between the ER and nER cohorts (Table 1).

When the RBC units transfused to patients in the nER cohort were stratified by ABO type, there were 1415 O-positive, 115 O-negative, 928 A-positive, 83 A-negative, 272 B-positive, 29 B-negative, and 7 AB-positive units. Similar to the ER cohort, differences in RBC unit age were also observed when the nER cohort was stratified by ABO type (Figure 3). The median age of an O-positive unit was 15 days and the median age of an O-negative unit was 11.5 days. A-positive, A-negative, and B-positive units had median ages between 14 and 19 days of age, while B-negative and AB-positive units had median ages of 31 and 27 days, respectively.

### 3.3. ER Cohort versus nER Cohort Comparisons

The median RBC unit age in the ER cohort was significantly greater compared to the median unit age in the nER cohort (20 [IQR 13, 30] versus 15 [IQR 7, 27] days, resp.; *P* < 0.001). However, it is important to note that both cohorts were within the same week of storage with an absolute difference of 5 days. A recent national survey reported that the mean storage age of a RBC unit transfused in the United States is 17.9 days [15]; this is consistent with the internal quality data at our institution (data not shown) as well as the current findings. Lastly, the majority of RBC units issued in both cohorts were type O (ER 81.4%, nER 53.7%) and the mean ages of these type O units from both cohorts fell within the third week of storage (ER 19.1 days, nER 15.7 days; *P* < 0.001). For non-type O RBCs, the mean ages of these units were significantly different between the two cohorts (ER 31.0 days, nER 20.1 days, *P* < 0.001).

### 3.4. Distribution of ABO Type Blood Products from the Regional Donor Population

Over the 30-year analysis period, 709,894 unique blood donors were identified. The distribution of ABO types of these donors and their respective products is shown in Table 2. As the data demonstrates, while type O donors represented the largest percentage of donors, the distribution of ABO types reflects that of a donor base in which Caucasian donors comprise the largest group of donors. Additionally, while the percentage of type O products used in the two cohorts was greater than the percentage of type O individuals present in the regional donor population, these differences did not achieve statistical significance (donors 44.1%, ER 37.4%, and nER 44.9%, *P* > 0.05).

## 4. Discussion

Although the effects of transfusing longer stored RBCs are currently unknown, most authors contend that if clinically significant impacts exist, they are most likely to occur with units stored for greater durations of time and usually greater than 28 days [1–6]. We demonstrate herein that RBC units issued to massively transfused patients within our large tertiary referral center in either an emergent or nonemergent fashion have ages that fall within the same week of storage. Although age differences between the two groups were statistically significant, an increase in storage duration of less than one storage week is unlikely to be of clinical significance, as even current randomized trials assessing outcomes based on RBC age have chosen to randomize groups separated by at least 8–12 days of storage [12, 16]. Review of the interquartile ranges and greatest length of storage indicates no disproportionate distribution of RBC unit age. Individuals for whom the MTP were activated did not receive older RBC units in any analysis except for the likely insignificant observation that they may receive between 1 and 4 units greater than 28 days. Together, these data suggest that massive transfusion, specifically MTP activation, does not result in the delivery of substantially older RBC units.

An interesting observation from this analysis was that ABO type influences the age of RBCs in patients who underwent massive transfusion. Analysis of type O RBCs revealed minimal differences between ER and nER groups, and since type O units constituted 63.3% of the total units transfused, this likely influenced the cumulative results. In both the ER and nER groups, the ages of non-type O RBCs issued were considerably older than type O units. Thus, in the setting of ER of non-type O units (in a scenario where a massively hemorrhaging patient had an available ABO typing), there was a significant increase in the mean age of RBC units issued, with all non-type O blood groups being over 30 days. Given the relative frequency with which RBCs are transfused in our busy tertiary referral Level I trauma center, it is likely that type O units do not reach older ages prior to their frequent utilization in these populations, whereas the other less frequently transfused non-type O units are more likely to be stored for longer durations prior to issue.

Our analysis of the frequency of donor ABO types from the regional blood donor center suggests that type O RBCs are not disproportionately available to our hospital relative to the use of these type O products in the ER and nER cohorts, further supporting the notion that these units are utilized more rapidly and thus would not be expected to reach ages as old as non-type O RBCs. In confirmation of these observations, when we compared the age of RBCs transfused to non-type O patients before and after the transfusion of type-specific units in the ER group, the median age of issued units increased by more than 2 weeks.

A possible explanation for the significantly younger ages of non-type O RBCs in the nER cohort could be unique RBC special need requirements based on differences in the patient populations between these two groups. As the nER group was composed of patients who were more likely to be on medical services, they would be expected to have a greater likelihood of requiring leukoreduced and/or irradiated RBCs, in addition to antigen-negative RBCs due to previous alloimmunization from transfusion or pregnancy, all of which take more time to acquire or prepare. Almost invariably, all of these special conditions are waived in the setting of ER of ≥10 RBC units, where the need for immediate transfusion in massive quantity exceeds the need for any unique RBC products. While only a hypothesis, it may be that those non-type O units which are leukoreduced, are irradiated, or lack certain antigens are in fact younger than units which are not manipulated, perhaps because these units are utilized more frequently and do not have an opportunity to reach older ages (similar to type O RBCs).

Our study has a number of limitations. The retrospective design does not control for all potential confounding variables. It is also important to note that this was a single-center analysis at a large tertiary academic medical center and the results might not be applicable to other institutions, particularly those that have different policies for issuing RBC units in emergency requests. Due to limitations in the available dataset, we were unable to calculate the difference in the average age of RBCs issued before and after receipt of type-specific blood for individuals with blood type O. However, in this study, as type O units were much younger than non-type O units, it is unlikely that there would have been as significant of an increase in RBC unit age in type O patients compared to the non-type O individuals. Finally, it is important to note that this study was not designed to assess outcome measures associated with RBC storage age. Rather, it was designed to be a descriptive analysis of the age of RBCs utilized during massive transfusion and the effects of ABO type on the age of units distributed. The clinical implications of storage age have yet to be determined in ongoing randomized trials [12, 16].

In summary, the anecdotal belief that massively transfused patients, specifically those for whom the MTP was activated, receive older RBC units is not supported by these data. However, when non-type O RBCs are utilized, the age of transfused units increases markedly and enters the range in which it is hypothesized that there may be a clinically significant effect of the storage lesion [11, 12]. Further research into RBC storage age and outcomes may consider ABO type analysis in their study designs.

## Figures and Tables

**Table 1:** Difference in number of transfused units of older ages between cohorts.

	ER	nER	*P* value	MTP	nMTP	*P* value
Total # of pts	**131**	**176**		**51**	**80**	
# (%) of pts who received ≥1 unit ≥28 days old	82 (62.6%)	120 (68.2%)	0.31	37 (72.5%)	45 (56.2%)	0.06
# (%) of pts who received ≥5 units ≥28 days old	32 (24.4%)	50 (28.4%)	0.44	18 (35.3%)	14 (17.5%)	0.02
# (%) of pts who received ≥1 unit ≥35 days old	54 (41.2%)	79 (44.9%)	0.52	24 (47.0%)	30 (37.5%)	0.28
# (%) of pts who received ≥5 units ≥35 days old	14 (10.7%)	25 (14.2%)	0.36	4 (13.7%)	7 (8.8%)	0.37

**Table 2:** Distribution of ABO type blood products from the regional donor population compared to ER and nER cohort RBCs. Data is presented as number (% of total).

	Donors	ER	nER
O	313276 (44.1%)	49 (37.4%)	79 (44.9%)
A	277461 (39.1%)	61 (46.6%)	71 (40.3%)
B	86009 (12.1%)	18 (13.7%)	23 (13.1%)
AB	33148 (4.7%)	0 (0.0%)	3 (1.7%)
Unknown∗	0 (0.0%)	3 (2.3%)	0 (0%)

Total	709,894 (100.0%)	131 (100.0%)	176 (100.0%)

*The blood type of 3 patients in the ER cohort was unable to be obtained in the available dataset.
